# Tannins Can Have Direct Interactions with Anthelmintics: Investigations by Isothermal Titration Calorimetry

**DOI:** 10.3390/molecules28135261

**Published:** 2023-07-07

**Authors:** Mimosa Sillanpää, Marica T. Engström, Petri Tähtinen, Rebecca J. Green, Jarmo Käpylä, Anu Näreaho, Maarit Karonen

**Affiliations:** 1 Department of Chemistry, University of Turku, FI-20014 Turku, Finland; mamsil@utu.fi (M.S.); mtengs@utu.fi (M.T.E.); peppe@utu.fi (P.T.); 2 School of Chemistry, Food and Pharmacy, University of Reading, Whiteknights, P.O. Box 224, Reading RG6 6AP, UK; rebecca.green@reading.ac.uk; 3 Department of Life Technologies, University of Turku, FI-20014 Turku, Finland; jakapy@utu.fi; 4 Department of Veterinary Biosciences, University of Helsinki, FI-00014 Helsinki, Finland; anu.nareaho@helsinki.fi

**Keywords:** anthelmintic, hydrolysable tannin, isothermal titration calorimetry, polyphenol, tannin-anthelmintic interactions, thiabendazole

## Abstract

Plant tannins are known for their anthelmintic and antiparasitic activities and have been increasingly studied to battle the ever-growing problem of anthelmintic resistance. While tannins have been shown to exhibit these activities on their own, one approach would be to use them as complementary nutrients alongside commercial anthelmintics. So far, research on the interactions between tannins and anthelmintics is limited, and few studies have reported both synergistic and antagonistic effects depending on the type of tannin and the method used. These interactions could either strengthen or weaken the efficacy of commercial anthelmintics, especially if tannin-rich diets are combined with anthelmintics used as oral drenches. To study these interactions, a series of hydrolysable tannins (HTs) was selected, and their direct interactions with thiabendazole (TBZ) were evaluated by isothermal titration calorimetry (ITC), which allowed the detection of the exothermic interaction but also the roles and significances of different structural features of HTs in these interactions. Our results show that HTs can have a direct interaction with the benzimidazole anthelmintic TBZ and that the interaction is strengthened by increasing the number of free galloyl groups and the overall molecular flexibility of HTs.

## 1. Introduction

Plant tannins are a class of specialized metabolites that are produced in plant tissues to protect the plant against various threats, for example, pathogens and herbivores, or biotic and abiotic stresses [1]. The quantities and types of tannins vary greatly between species and even between individuals or plant parts of the same species. Tannins can be divided into three major groups: proanthocyanidins (PAs, syn. condensed tannins), hydrolysable tannins (HTs), and phlorotannins. From these, PAs and HTs possess large structural variability; PAs are oligo- and polymers formed of two or multiple flavan-3-ol units, while HTs are a diverse class of monomeric and oligomeric polyphenols with monomeric units consisting of a cyclic or acyclic polyol at their center furnished with a variable number of esterified variants of galloyl groups [2]. PAs and HTs have been shown to possess a number of different bioactivities, such as antioxidant [3,4,5], antimicrobial [6,7,8], and antiparasitic [9,10,11,12,13] activities. They have also been proven to produce a number of beneficial effects on ruminants when added to animal feed, including reduced greenhouse gas emissions and increased milk production [14,15,16].

The anthelmintic activities of tannins are of interest to scientists, as anthelmintic resistance has been on the rise ever since the development and increased use of both narrow- and broad-spectrum anthelmintics such as benzimidazoles and macrocyclic lactones [17,18,19]. Resistance to anthelmintics not only affects the health of the host animal but also substantially affects the productivity and income of the whole agribusiness [20]. Moreover, anthelmintic resistance has been observed to develop relatively quickly after introducing a new anthelmintic substance to a nematode population in less than ten years [21,22]. It warrants more sustainable solutions alongside more traditional helminth control strategies. The anthelmintic potential, structural variability, and sustainable availability, along with other beneficial effects, make tannins an appealing tool to battle this growing problem, either on their own as tannin fractions or tannin-rich feed or as complementary nutraceuticals added to other animal feeds [14,23]. The latter approach has gained more interest as natural compounds, such as monoterpenes and PAs, have been shown to increase the efficacy of anthelmintics such as levamisole, albendazole, and ivermectin [24,25,26,27,28,29]. However, the opposite effect has also been shown, as some tannins have been linked to decreasing the anthelmintic efficacy of ivermectin [30]. At the moment, it is still unknown whether these effects are synergistic or antagonistic and whether they are the results of direct or indirect modes of action, as the interactions have been mainly studied through in vivo and in vitro experiments and not on a molecular level. For example, polyphenols could indirectly potentiate the effect of an administered anthelmintic via phospho-glycoprotein (P-gp) modulation, as P-gp modulators have been shown to potentiate the efficacy of ivermectin [29,31], while more direct modes of action could include increased membrane permeability as a result of complexation [32].

**Figure 1:**
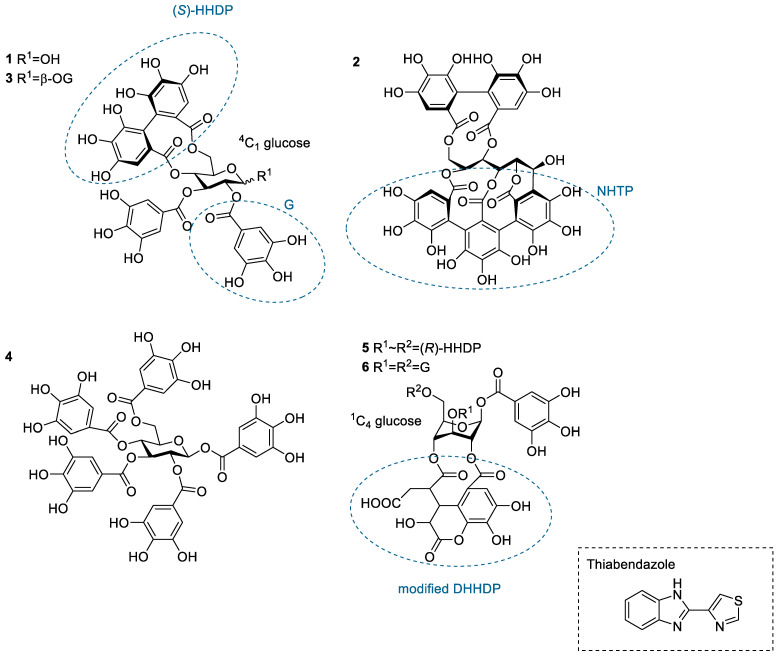
Structures of the studied hydrolysable tannin monomers: tellimagrandin I (**1**), vescalagin (**2**), tellimagrandin II (**3**), 1,2,3,4,6-penta-*O*-galloyl-β-d-glucose (**4**), chebulagic acid (**5**), and chebulinic acid (**6**). The anthelmintic used in the study, thiabendazole, is also depicted. DHHDP, dehydrohexahydroxydiphenoyl group; G, galloyl group; HHDP, hexahydroxydiphenoyl group; NHTP, nonahydroxytriphenoyl group.

**Figure 2:**
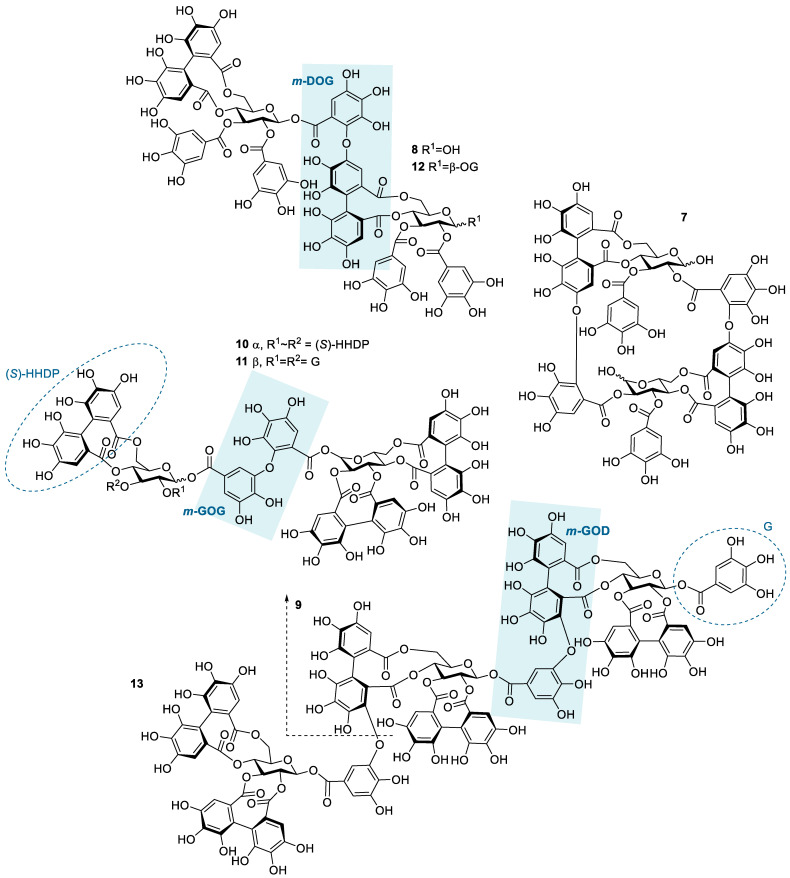
Structures of the studied hydrolysable tannin dimers and trimers: oenothein B (**7**), rugosin E (**8**), sanguiin H-6 (**9**), agrimoniin (**10**), gemin A (**11**), rugosin D (**12**), and lambertianin C (**13**). Examples of different types of linkages between monomeric units are highlighted by blue shading; *m*-DOG (valoneoyl group), *m*-GOG (dehydrodigalloyl group), and *m*-GOD (sanguisorboyl group). In the abbreviations, the first letter represents the oxygen donor, and the last letter is its acceptor. D, HHDP, hexahydroxydiphenoyl group; G, galloyl group; O, oxygen.

While direct interactions between monoterpenes and anthelmintics have been proven [24], our knowledge still lacks a more systematic overview of tannin-anthelmintic interactions. Isothermal titration calorimetry (ITC) is a label-free method traditionally used to observe binding between a ligand and a macromolecule, i.e., interactions between enzymes and their ligands. During a typical ITC measurement, a ligand solution is titrated into a macromolecule solution, and the instrument detects the reaction heat caused by the ligand-macromolecule interaction. From an ITC measurement, it is possible to obtain the thermodynamic binding parameters of the interaction, such as binding affinity (*K_a_*), enthalpy (Δ*H*), and binding stoichiometry (*n*). In tannin analytics, ITC has previously been used to measure the binding of tannins to different proteins and lipids [33,34,35,36,37,38]. Additionally, the binding of flavan-3-ols from tea to risperidone has been studied via ITC [39]. In this study, thiabendazole (TBZ, Figure 1) was used as a model substance as benzimidazoles are a widely used drug class, and TBZ has good solubility in buffer systems used in ITC. A series of HTs (Figure 1 and Figure 2, Table S1) was selected as they could be easily purified as individual compounds, which allowed the evaluation of their structure-activity relationships in detail. ITC was utilized to investigate whether HTs can have direct interactions with TBZ, what the thermodynamics of the interaction is, and how different structural features of plant HTs affect the interaction strength.

## 2. Results and Discussion

### 2.1. ITC Method Development for HT-Anthelmintic Interactions

During the ITC method development, a wide range of pHs, DMSO percentages, and sample concentrations were tested. After solubility testing, a 3 mM TBZ solution in pH 3.6 citrate buffer with 5% DMSO was used in the experiments. The TBZ solution was prepared by first dissolving the TBZ in DMSO to form a 60 mM stock solution, then diluting the stock solution with the citrate buffer to form the final 5% DMSO solution. In general, the DMSO percentage should be kept to a minimum in ITC measurements, as a percentage too high can affect the baseline of the titration. The usual recommended amount of DMSO should be in the range of 1–2%, with a recommended upper limit of 10% [40]. A titration temperature of 40 °C was chosen as the increase in temperature yielded better saturation of the available binding sites at the end of the isotherm and is close to the physiological temperatures of sheep and cattle (about 39 °C [41]). On top of this, different technical parameters were tested, such as injection volume, reference power (RP), and feedback modes of the instrument (low or high gain). The resulting method is a compromise; a smaller RP resulted in a more distinct shape for the isotherm, and better saturation was reached at the end of the titration in comparison to a higher RP (Figure S1), but this RP value would not be applicable for possible stronger interactions within the sample set. Thus, an RP of 5 µCal/s was used, which is generally considered a good value for unknown reactions [42].

Ideally, during the ITC method development, the concentrations of the interacting compounds are chosen so that the resulting isotherm has a sigmoidal shape. To achieve this, the concentrations of the ligand and macromolecule solution should be carefully chosen, and a commonly used term, the Wiseman parameter c [43], should be in the range of 10–500. In an ideal measurement, during the first injections, all the interaction sites of the titrant are filled, and at the end of the titration, all the sites of the analyte in the sample cell are filled. However, this requires adequate interaction strength to be achievable. If the interaction strength is low, the resulting parameter c is usually also low, as the concentrations of the samples are the next limiting factor. This is usually a consequence of solubility or sample availability problems, as was also the case in our study. For the studied HT monomers, we were able to observe direct interactions and obtain qualitative information about the effects of different structural characteristics of the tannins used, even though thermodynamic binding parameters could not be obtained. The raw data and resulting isotherms of the monomers are presented in the Supplementary Materials in Figure S2. For the HT dimers and the trimer, the interactions were stronger, but the data in its current form could not be fitted without uncertainty due to non-ideal titration conditions in terms of the parameter c [43] (Figures S3 and S4). Except for the HT dimer, rugosin D, with the strongest interaction with TBZ, we performed initial thermodynamic analysis, i.e., fitting of the isotherm, to obtain the thermodynamic binding parameters (*K_a_*, *n*, Δ*H*) of the interaction. The fit and the corresponding thermodynamic parameters are included in the Supplementary Materials in Figure S5, as discussed later in Section 2.3.

**Figure 3:**
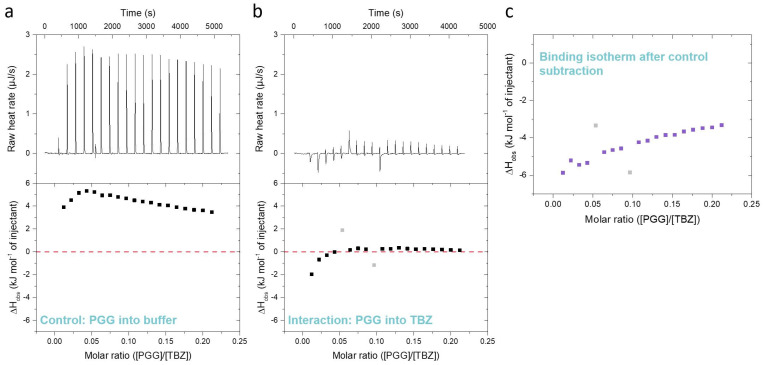
Differences between (**a**) a control titration of pentagalloylglucose (PGG) into 5% DMSO citrate buffer, (**b**) an actual interaction titration of 3 mM PGG into 3 mM thiabendazole (TBZ) in 5% DMSO citrate buffer, and (**c**) the resulting isotherm obtained by subtracting the control data from the data of tannin into TBZ. The outliers marked in light gray are caused by baseline fluctuations during the titration.

Furthermore, during the ITC method development, it is very important to note that tannins are known to self-aggregate in concentrated solutions due to their hydrophobicity [35,36,44]. This is also the case in the ITC titrations, and when a concentrated tannin solution is injected into the analyte solution in the sample cell, the tannins deaggregate due to dilution in an endothermic reaction [35]. The degree of deaggregation is dependent on the amount of tannin already present in the sample cell; therefore, successive injections of tannin into the sample cell gradually lead to lower enthalpy changes (Figure 3a), as also shown in previous studies [33,35,36,45]. For the iTC200 instrument used in this study, the endothermic reaction is perceived as large upward-pointing peaks, as shown for a control titration of tannin into a buffer in Figure 3a. However, the interaction between the tannin and TBZ is an exothermic reaction resulting in peaks pointing downwards, as presented in Figure 3b. As endothermic deaggregation also occurs during the titration of the tannin into a TBZ solution, removing the control data from the tannin into buffer measurement data is a critical step during the data processing. During the processing of the ITC data, the peaks of each injection of the control experiment and the actual titration of the tannin into TBZ are first integrated (Figure 3a,b, raw heats (µJ s^−1^)), after which the observed molar enthalpy changes of each injection (kJ mol^−1^) from the control experiment are calculated and subtracted from the corresponding values of the actual tannin into TBZ experiment, resulting in an isotherm depicting only the observed enthalpy changes from the interaction between the tannin and TBZ (Figure 3c).

**Figure 4:**
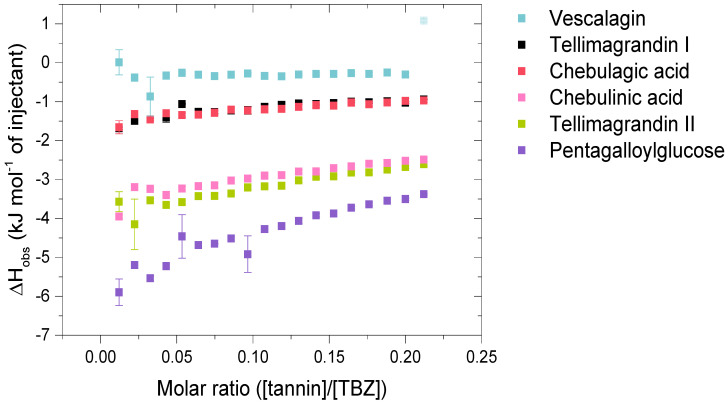
Isotherms of the interactions of hydrolysable tannin (HT) monomers with thiabendazole (TBZ) depict the released heat as kJ per one mole of injectant as a function of the molar ratio of tannin to TBZ. Standard errors between three replicates are also shown (*n* = 3). Outliers with small standard errors (like the last injection marked in a lighter color for vescalagin) are caused by baseline fluctuations in the control titration.

**Figure 5:**
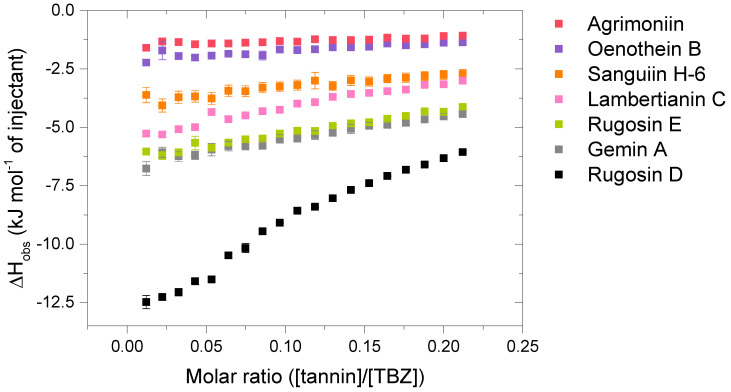
Isotherms of the interactions between the analyzed hydrolysable tannin (HT) dimers and the HT trimer, lambertianin C, and thiabendazole (TBZ). Standard errors between three replicates are also shown (*n* = 3).

In theory, the analyte, or in this study, the anthelmintic, present in the sample cell might also affect the deaggregation process of the injected tannin, especially when the sample concentrations are as high as in our experiments. These effects on tannin deaggregation could be caused, for example, by the presence of the anthelmintic-anthelmintic interaction or by the interactions between the tannin aggregates and the anthelmintic. These complex processes could also be taking place in our data, as it can be seen from Figure 3, Figure 4 and Figure 5 that the heat signals do not approach zero at the end of the titration despite the available binding sites becoming saturated, as in the case of PGG, for example. This can also be attributed to non-ideal titration conditions, as the same method was used for multiple tannins with different interaction strengths instead of optimizing it separately for each tannin. However, these additional processes taking place during the titration cannot be measured without also observing the interaction heat itself. The large observed heats from the tannin deaggregation could have been refrained mainly by titrating the anthelmintic into the tannin solution, but this approach was not applicable in our study due to limitations in solubility (see Section 3.4). Moreover, previous ITC studies on the interactions between different HTs and proteins or lipids [33,36,37,44,45] have all been conducted by titrating tannins into the studied macromolecule. Therefore, this experimental setting of titrating tannin into the anthelmintic made comparing the previous results easier. In addition, it enables future studies on the combined interactions between tannins, macromolecules, and anthelmintics.

### 2.2. Interactions between Tannin Monomers and Thiabendazole

The HT monomers used were tellimagrandin I (**1**), vescalagin (**2**), tellimagrandin II (**3**), 1,2,3,4,6-*O*-d-pentagalloylglucose (PGG, **4**), chebulagic acid (**5**), and chebulinic acid (**6**). This series allowed the comparison of different structural variabilities of HTs: the overall flexibility of the structure, the presence and number of free galloyl groups versus hexahydroxydiphenoyl groups (HHDP), acyclic versus glucopyranose cores, and the effect of a modified dehydrohexahydroxydiphenoyl group (DHHDP), also known as the chebuloyl group (Figure 1). In general, the overall flexibility of an HT is decreased by the linkage of two free galloyl groups into one HHDP group and by replacing the glucopyranose core with an acyclic glucose that is attached to a nonahydroxytriphenoyl group (NHTP). In the HT series used, vescalagin (HHDP + NHTP groups) and chebulagic acid (HHDP + DHHDP + galloyl groups) have the most rigid structures, while PGG (5 × galloyl groups) has the most flexible structure. The isotherms of the analyzed HT monomers are shown in Figure 4, from which we can see that the monomers with the weakest interactions or no affinity to TBZ were tellimagrandin I, chebulagic acid, and vescalagin, while higher affinities were measured for chebulinic acid, tellimagrandin II, and PGG. Overall, the affinity of HT monomers to TBZ was detectable but weak, with the observed changes in enthalpy per mole of injectant ranging from around –6 to 0 kJ/mol (Figure 3c and Figure 4). For comparison, much stronger interaction enthalpies (−40–0 kJ/mol) between tannins and a widely used model protein, bovine serum albumin (BSA), have been observed [45]. However, the interaction strengths were strong enough to allow us to qualitatively compare the effect of tannin structure to the observed affinity for TBZ.

The effect of the number of galloyl groups in the HT structure on the interaction with TBZ is seen when comparing tellimagrandins I and II; the only structural difference is the presence or absence of a galloyl group in the anomeric position of the glucopyranose ring. Tellimagrandin II, with the additional galloyl group, has a significantly stronger interaction with TBZ than tellimagrandin I. For tellimagrandin II and PGG, the difference is that the two free galloyl groups in positions 4 and 6 of the glucopyranose core in PGG are replaced by an HHDP group in tellimagrandin II, limiting the flexibility of the latter structure and thereby also weakening the interaction strength in comparison to PGG (Figure 4). Similar structural differences can be found between chebulagic and chebulinic acids (Figure 1). Chebulinic acid with two free galloyl groups in the structure has a stronger interaction with TBZ than chebulagic acid with an HHDP group (Figure 4). From the whole set of monomers, PGG exhibited the strongest interaction with TBZ, with its five free galloyl groups and the most flexible structure. This emphasizes the importance of both the presence and the number of free galloyl groups in the interaction with TBZ.

From Figure 4, it can be observed that vescalagin had no detectable interaction with TBZ at the concentrations used. This is most likely caused by the rigidity of the structure due to the presence of NHTP and HHDP groups. The result is in accordance with the literature regarding interactions between vescalagin and macromolecules [9,45]. The other monomers with weaker affinity, tellimagrandin I and chebulagic acid, exhibited small but detectable changes in enthalpies. Interestingly, these two tannins resulted in nearly identical Δ*H*, even though tellimagrandin I has an additional free galloyl group compared to chebulagic acid and is more flexible than chebulagic acid. The similar interaction strengths might result from the different conformations of the glucopyranose cores or the difference in the positioning of the galloyl groups: chebulagic acid has its only free galloyl group in the anomeric position of its ^1^C_4_ glucose core, where it is sterically less hindered in comparison to the galloyls in the 2 and 3 positions of the ^4^C_1_ glucopyranose core of tellimagrandin I. Another factor might be the other differences in the structures, as tellimagrandin I has an HHDP group and chebulagic acid has an HHDP and a chebuloyl group. This is less likely to be the case as tannins with the same number of free galloyl groups but with either an HHDP or a chebuloyl group demonstrate similar interaction strengths, as seen in tellimagrandin II and chebulinic acid. However, the interaction strengths observed for tellimagrandin I and chebulagic acid were relatively small, making it challenging to make a decisive structural comparison between the two.

### 2.3. Interactions of Hydrolysable Tannin Dimers and a Trimer to Thiabendazole

The HT dimers studied were oenothein B (**7**), rugosin E (**8**), sanguiin H-6 (**9**), agrimoniin (**10**), gemin A (**11**), and rugosin D (**12**). A trimeric derivative of sanguiin H-6, lambertianin C (**13**), was also included in the sample set. This HT series (Figure 2) allowed the comparison of the effects of similar structural features as within the monomeric series (HHDP groups versus galloyl groups and the role of a variable number of galloyl groups in the structure) and also the effects of the increase in molecular weight and structural flexibility, as for oligomeric HTs, the flexibility is also affected by the linkage types between the monomeric units. The possible linkage types for HT oligomers are GOG (dehydrodigalloyl), DOG (valoneoyl), GOD (sanguisorboyl), D(OG)_2_, and *C*-glycosidic type [46], where G stands for a galloyl group, O for oxygen, and D for an HHDP group, as shown in Figure 2. In these abbreviations, the first letter represents the functional group that acts as an oxygen donor, while the last letter represents the oxygen acceptor. In our sample set, *m*-DOG, *m*-GOG, and *m*-GOD linkages are represented. 

Compared to the observed enthalpy changes of the interactions of HT monomers with TBZ (Figure 4), the dimers and the trimers showed generally larger Δ*H* values (Figure 5). However, the differences between the observed enthalpies were smaller than expected, as for tannins, the increase in the degree of oligomerization and molecular weight has previously been linked to increased interaction strength with the studied macromolecule [36,44]. From a structural perspective, the increase in the degree of oligomerization would provide a larger number of binding sites for the small TBZ to bind to. However, this was not the case for all the HT dimers, as the interactions of oenothein B and agrimoniin with TBZ exhibited distinctively smaller enthalpy changes in comparison to the HT monomers with higher affinity to TBZ (Figure 4 and Figure 5). For oenothein B, the rather small affinity for TBZ can be linked to its rigid macrocyclic structure, which may also cause steric hindrance for the two galloyl groups in the structure (Figure 2). For agrimoniin, the result was more unexpected, as even though there are no free galloyl groups but instead four HHDP groups in the compound, the structure still has some flexibility because of the flexible *m*-GOG linkage between its two monomeric units. The observed molar enthalpy changes of these two HT dimers were of similar magnitude when compared to the enthalpy changes of the monomeric tellimagrandin I and chebulagic acid, which both have free galloyl groups in their structures. 

By using the pair of a dimer and its corresponding trimer, sanguiin H-6 and lambertianin C (Figure 2), the effect on the interaction strength due to the increase in the molecular weight can be investigated. Both compounds have only one free galloyl group, and their monomeric units are linked via *m*-GOD linkages, which are relatively hindered and therefore make the structures reasonably rigid. Higher interaction heats were observed for lambertianin C than for sanguiin H-6, but in general, the observed heats for lambertianin C were surprisingly small, even smaller than the heats for three of the other HT dimers in the sample set (Figure 5). From this, it can be deduced that while the interaction strength follows the general trends observed for other tannin interactions, such as the increase in strength due to the increasing number of free galloyl groups and molecular flexibility, the effect of molecular weight on its own is not emphasized. The relatively weak interaction with TBZ may also be linked to the strong self-association observed for lambertianin C, as previously demonstrated [37]. The slight increase in the released heat from sanguiin H-6 to lambertianin C could result from the increase in the number of aromatic hydroxyl groups within the additional monomeric unit in the latter (29 OH groups in sanguiin H-6 versus 43 OH groups in lambertianin C) or simply due to the additional polyphenolic rings in the two HHDP groups in the latter. 

Although the increase in interaction strength is small when comparing dimeric sanguiin H-6 and trimeric lambertianin C, the increasing degree of oligomerization slightly increases the interactions. A larger enthalpy difference was observed for dimers rugosin E and D (which are formed of monomeric tellimagrandins II + I and tellimagrandins II + II linked via *m*-DOG, respectively) than for the corresponding monomers (Figure 4 and Figure 5). From this, it can be deduced that while the degree of oligomerization affects the interaction strengths between tannin and TBZ, the functional groups of the monomers forming the oligomers are also important. For example, when comparing the functional groups of agrimoniin and sanguiin H-6, the increase in Δ*H* can be linked to the additional free galloyl group in the structure of sanguiin H-6.

Interestingly, gemin A and rugosin E exhibited similar changes in enthalpies for their interaction with TBZ, even though rugosin E has four free galloyl groups while gemin A only has two (Figure 2 and Figure 5). The reason for this can be attributed to the difference in structural flexibility, as the monomeric units in gemin A are attached via *m*-GOG linkage, whereas for rugosin E, the linkage type is a more rigid *m*-DOG (Figure 2). When comparing agrimoniin A and gemin A, a similar effect can be seen for the monomers chebulagic acid and chebulinic acid: when there are two free galloyl groups instead of one HHDP group, the interactions with TBZ become stronger. Gemin A and rugosin E both interact with TBZ stronger than lambertianin C, highlighting the importance of molecular flexibility and free galloyl groups even further. 

The largest interaction heat from the whole sample set was measured for rugosin D, which is in accordance with previously highlighted trends as rugosin D has five free galloyl groups and is thus relatively flexible, despite having one HHDP group and the monomeric units linked via *m*-DOG (Figure 2). When comparing the structures of rugosin E and rugosin D, the only difference is the addition of one galloyl group in the anomeric position of the glucose core, and, as seen from the results, this has a significant effect on the strength of the interaction with TBZ. A similar effect was also observed for tellimagrandins I and II, as there, the released heats were roughly doubled. For rugosin D, we were able to fit the data and perform an initial thermodynamic analysis to obtain rough estimates of *K_a_* and *n* (Figure S5). The obtained *K_a_* (1365 M^−1^) can be considered the maximum value for the binding affinity that the used HTs can have with TBZ at equilibrium, and the parameter *n* (0.180) indicates that multiple TBZ molecules bind to one tannin molecule, as suspected from a structural point of view. 

### 2.4. Structural Features and Bioactivities of HTs Associating with Their Interactions with TBZ 

Overall, the interaction patterns of tannins with TBZ followed similar trends as have been recorded for other types of tannin interactions, such as tannin-protein or tannin-lipid interactions [37,44,45,47]. These structural characteristics favorable for the interaction with TBZ were the number of free galloyl groups and overall molecular flexibility. The effect of the free galloyl group attached to the anomeric position of the glucopyranose core was highlighted. Interestingly, the increase in molecular weight did not substantially affect the interaction strength, which is a deviation from the trends usually associated with tannin interactions. The effect of either an HHDP group or a chebuloyl group on the interaction was similar. 

Given the structural characteristics of the studied anthelmintic TBZ, i.e., its potential sites for hydrogen bonding and π–π stacking, interactions with tannins through these mechanisms were anticipated. However, even though HHDP groups offer an equal number of binding sites for these reaction mechanisms as galloyl groups do and thus are expected to interact with the anthelmintic, they showed little interaction with TBZ. This lack of interaction may be attributed to the steric hindrance caused by the antiparallel C–C linked galloyl units in the HHDP and NHTP groups. Therefore, the π electrons of the aromatic groups in TBZ cannot optimally approach the π electrons of the galloyl subunits in an HHDP to form the required hydrophobic interactions. The smaller amount of molecular freedom in the structure of the tannin was thought not to be a hindrance for the smaller TBZ, but this hypothesis proved to be incorrect, as seen when comparing interactions of HT monomers, dimers, and the trimer with TBZ. These findings suggest that free galloyl groups are required for the interaction of HTs with TBZ, and their availability is paramount for these hydrophobic interactions to occur. Silva et al. [24] investigated the sites of interaction of two different anthelmintics, albendazole, and levamisole, with the monoterpenes r-carvone and s-carvone and attributed the observed synergistic effects to strong interactions with the carbonyl and amine groups of the anthelmintics. Evidence of hydrogen bonding in the interactions of albendazole with the monoterpenes was also found. Although the structures of these anthelmintics differ from those of TBZ used in this study, they verify the possible interaction mechanisms described above.

HTs have been shown to exhibit anthelmintic activity themselves by inhibiting the egg hatching and motility of L1 and L2 stage larvae of *Haemonchus contortus* and the exsheathment of *Haemonchus contortus* and *Trichostrongylus colubriformis* L3 larvae in vitro [9,13]. In these studies, clear relationships have been observed between HT structures and their anthelmintic activities. The influence of HT structure on the interaction strengths in our study follows similar trends as found earlier [9,13]; the number of free galloyl groups, molecular flexibility, and degree of oligomerization of HTs, on average, had a positive effect on the anthelmintic activity, while the activity decreased when two galloyl groups merged into one HHDP group. The linkage unit *m*-GOD, present in sanguiin H-6 and lambertianin C, has been found to play a role in the increase of anthelmintic activity, while in our data, the effect of the linkage unit seemed minor and was overshadowed by the number of free galloyl groups. This can be explained by the different targets for the interaction, as during the anthelmintic action of tannins, they can bind to different proteins in the sheath structure of the nematodes [13], while in our study, the target of interaction is smaller, and multiple TBZ molecules bind to one tannin molecule, inversely to tannin-protein interactions [36,45], increasing the need for molecular flexibility for the interaction. Rugosins E and D are the most active ones in inhibiting the larval exsheathment [9], and they also had the highest affinity for TBZ in this study. This opens up interesting perspectives for further research, considering the possible use of tannins as complementary additives alongside commercial anthelmintics. In vivo, tannins are not fully soluble, and they can be bound to other macromolecules, such as proteins [35,47,48], lipids [37,49,50], and fibers [51,52,53], to an increasing extent, for example, during feed production or eating. These tannin-macromolecule interactions are also affected by the same structural features of HTs. Therefore, a detailed understanding of these interactions and their mechanisms in a combinatorial manner is very important when the beneficial bioactivities of tannins are evaluated. 

As tannin-anthelmintic interactions for HTs and TBZ were proven in this study, future research targets should be expanded to other anthelmintics as well as proanthocyanidins and include studies to confirm the mechanisms and sites of the interaction and to see if the complexation of these compounds affects the bioavailability of TBZ or other anthelmintics. These targets could be approached by NMR spectroscopy, molecular modeling, and docking studies. After identifying the mechanisms of interaction, in vitro studies could be conducted with selected tannins and different commercial anthelmintics to study how the bioactivities of tannins affect the combinatorial effect of the tannin and the anthelmintic. Lastly, the animal feeds, including selected HTs, should be tested in vivo to see the possible indirect modes of action [25,26,29,31,54] and if a mixture of tannins affects the whole process differently, as tannins, along with other natural compounds, have been shown to exhibit synergistic effects [55,56]. In addition, as mentioned above, tannins are known to bind to other macromolecules present in the gut, for example, dietary proteins or polysaccharides, and their affinities to tannins should be taken into account, as tannins most probably will not selectively interact with TBZ in vivo.

## 3. Materials and Methods

### 3.1. Reagents

Thiabendazole (CAS 148-79-8, PESTANAL^®^, analytical standard, ≥98.0%), dimethyl sulfoxide (DMSO, CAS 67-68-5, ≥99.9%), and buffer reagents sodium citrate dibasic dihydrate (CAS 6132-04-3, ≥99.0%) and citric acid monohydrate (CAS 5949-29-1, ≥99.0%) were all purchased from Sigma-Aldrich International GmbH, St. Louis, MO, USA. Reagents used for the extraction, purification, and identification were analytical grade acetone and methanol (≥99%, Sigma-Aldrich, France); HPLC grade methanol (≥99.9%, Sigma-Aldrich, France); acetonitrile (≥99.9%, Honeywell, Seelze, Germany); and formic acid (99–100%, VWR Chemicals, Rosny-sous-Bois-cedex, Paris, France); ethanol absolute (≥99.8%, VWR International, Fontenay-sous-Bois, Paris, France); LC-MS grade acetonitrile (Honeywell, Seelze, Germany); and LC-MS grade formic acid (VWR International, Fontenay-sous-Bois, Paris, France). The water used was type I ultrapure water prepared with the Merck Millipore Synergy UV system (Merck KGaA, Darmstadt, Germany).

### 3.2. Collection of Plant Material, Extraction, and Purification

The collection of plant material, extraction, and purification of individual compounds was mainly conducted as previously reported [2,4,36,45,57,58,59,60]. Fresh plant material was collected directly into 1 L glass bottles, and the bottles were filled with acetone. The material was extracted multiple times with 80/20 acetone/water (*v*/*v*). After ensuring proper extraction of the target compounds, all the extracts were combined, the organic solvent was evaporated, and the aqueous extracts were frozen and lyophilized. The crude extracts were dissolved in water and then fractionated by Sephadex LH-20 column chromatography using a similar elution protocol as previously reported, but the eluents, water, aqueous methanol, and aqueous acetone, were adjusted depending on the ET to be isolated [2]. After the first fractionation by Sephadex LH-20, the obtained HT-rich fractions were fractionated further by preparative HPLC and then purified via semipreparative HPLC. PGG was prepared from commercial tannic acid via methanolysis [61]. All the steps were monitored by UHPLC-DAD-ESI-MS/MS (Section 3.3). Original plant species and materials, purities of studied HTs, their exact and calculated molecular masses, mass errors, and characteristic fragmentation patterns used for identification are described in more detail in the Supplementary Materials in Table S1 [2,4,57,59,60,62,63,64,65]. These HTs have been previously characterized by NMR spectroscopy by Virtanen et al. [37].

### 3.3. UHPLC-DAD-ESI-HRMS and UHPLC-DAD-ESI-MS/MS Analyses

The HTs were characterized using an Acquity UPLC system (Waters Corp., Milford, MA, USA) connected to Q Exactive Orbitrap^TM^ (Thermo Fisher Scientific GmbH, Bremen, Germany). The instrument used to determine the purities of HTs was a similar UPLC system connected to a Xevo TQ triple-quadrupole mass spectrometer (Waters Corp., Milford, MA, USA). 

The column used for both analyses was an Acquity UPLC BEH Phenyl column (2.1 × 100 mm, 1.7 µm, Waters Corp., Wexford, Ireland) with a column temperature of 40 °C. Acetonitrile (A) and 0.1% HCOOH (B) were used as eluents with a flow rate of 0.5 mL min^−1^. The elution profile was as follows; 0–0.5 min: 0.1% A (isocratic gradient); 0.5–5 min: 0.1–3% A (linear gradient); 5–6 min: 30–35% A (linear); 6.0–6.1 min: 30–90% A (linear); 6.1–9.5 min: 90–0.1% A (column wash and stabilization). The injected sample volume was 5 µL. Negative ionization was used to collect the HRMS data; a heated ESI source was used with a spray voltage of −3.0 kV, a sheath gas (N_2_) flow rate of 60, an auxiliary gas (N_2_) flow rate of 20, a sweep gas flow rate of 0, a capillary temperature of +380 °C, and an in-source collision-induced dissociation (CID) of 30 eV. For full scan MS, the mass range for orbitrap was *m/z* 150–2250, the resolution 35,000, and the automatic gain control 3 × 10^6^. For the MS/MS analyses, a TopN method with the following parameters was used: the stepped normalized collision energies of 20, 50, and 80 eV, the resolution of 17,500, and the automatic gain control of 1 × 10^5^. Prior to analysis, the Orbitrap^TM^ was calibrated using Pierce ESI Negative Ion Calibration Solution (Thermo Fisher Scientific Inc., Waltham, MA, USA). The data was processed using Thermo Xcalibur Qual Browser software (Version 4.1.31.9, Thermo Fisher Scientific Inc., Waltham, MA, USA). Before the analysis, samples were dissolved in 10% aqueous ethanol and filtered through 0.2 µm PTFE filters. 

For the determination of purities, a negative ionization mode was also used, and the corresponding ESI parameters were as follows: capillary voltage 1.8 kV, desolvation temperature 650 °C, source temperature 150 °C, and desolvation and cone gas (N_2_) flow rates of 1000 and 100 L/h, respectively. The MS method consisted of a full scan analysis with a mass range of *m/z* 150–2000 and group-specific multiple reaction monitoring methods as previously described [66]. Sample preparation was performed as described above. 

### 3.4. Isothermal Titration Calorimetry

Calorimetric analyses were conducted with the MicroCal iTC200 (Malvern Panalytical Ltd., Malvern, UK). Measurements were started through method development and testing. The studied TBZ has limited solubility, so we tested different buffers, solvent pHs, and DMSO concentrations. Solubility tests were conducted with two different buffers: citrate buffer and phosphate buffer. No noticeable difference was found between these two, and as there are tannin stability issues associated with the phosphate buffer [37], citrate buffer was selected for further testing. TBZ was more soluble in lower pH ranges of 3.0–3.6. DMSO concentrations of 2–10% were tested; a higher amount than 10% DMSO is not generally recommended for ITC measurements, as it might affect the baseline of the titration. The other important factor was to adjust the concentrations used so that the amount of purified tannins needed was reasonable. Based on previous studies using tannins in ITC [33,35,36,44,45], the method development was started with the idea of titrating tannin to the anthelmintic, even though because of its size, TBZ would be the ligand in the possible interaction. Changing the titrant from tannin to anthelmintic was also tested, but no detectable heat was observed at the concentrations used.

The model tannin used in the method’s development was PGG. The testing started with titrating 50 µM PGG into 200 µM TBZ, and concentrations and other parameters were gradually changed from there to form the final method. First, we narrowed down the concentrations of PGG and TBZ, where we could detect heat released from the interaction, and then continued towards improving the shape of the gained isotherm by adjusting the concentrations and other parameters. Different injection volumes (1.3–2 µL) and their effects on the thermogram were also tested. Increasing the experiment temperature from 25 °C to 40 °C was noticed to enhance the saturation of the binding sites of TBZ at the end of the isotherm, so an experiment temperature of 40 °C was chosen. Reference power (RP) values of 2, 3, 5, and 7 were also tested, and an RP of 5 µCal/s was found to be most suitable for different types of tannins. Different feedback modes, which affect the instrument response time and sensitivity, were also tested, and a high gain mode was chosen.

For the final method, each experiment consisted of a background measurement of 3 mM tannin in the buffer and three replicates of 3 mM tannin in 3 mM TBZ. Heats from injecting buffer into buffer and buffer into TBZ were also measured, but they were minor and remained consistent throughout the titration sequence, so they were not included in the final data processing. The experiment temperature was 40 °C. The injection volume of the first injection was 0.4 µL, followed by the titration of tannin into TBZ in 19 × 2 µL aliquots. The first injection was not included in the data processing, as the heat released from the first injection is lower due to a small amount of injectant dissolving from the tip of the injection syringe into the titrant during the equilibration period at the beginning of the analysis. The initial delay before the first injection was 400 s, the spacing between injections was 250 s, and the stirring was set to 750 rpm. An RP of 5 µCal/s was used. A water-to-water titration and EDTA test kit provided by the manufacturer were used to monitor the state of the instrument and ensure the cleanliness of the sample cell. 

Prior to analysis, TBZ was first dissolved in pure DMSO to form a 60 mM stock solution. This stock was then diluted 20-fold with a pH 3.6 citrate buffer to form a 3 mM TBZ solution with 5% DMSO. Tannins were dissolved into a similar solution, i.e., 5% DMSO in citrate buffer, to prevent additional heat from the buffer mismatch during titration. The only exception was chebulinic acid, for which 10% DMSO had to be used due to solubility issues. The DMSO-% for the TBZ solution was matched accordingly by preparing a 30 mM stock solution, which was diluted 10-fold. 

Raw data from the microcalorimeter was processed with NanoAnalyze (v. 3.12.0, 2008, TA Instruments, New Castle, DE, USA). During the data processing, all the injection heats were integrated, and the heats from the background measurement (tannin to buffer) were subtracted from the heats of the tannin to TBZ measurements. After background removal, isotherms depicting the heat released (kJ) per mole of injectant as a function of the molar ratio of tannin to TBZ were obtained and, if possible, fitted with a binding model for independent binding sites. Each of the three replicates was fitted independently. 

## 4. Conclusions

In this study, we successfully developed an ITC method to analyze, for the first time to our knowledge, the direct interactions of tannins with a benzimidazole anthelmintic, TBZ. Our carefully selected HT series revealed that the interaction with TBZ is strongly affected by the free galloyl groups, molecular flexibility, and rotational freedom in the tannin structure. The interaction is also greatly strengthened by a free galloyl group attached to the anomeric position of the glucopyranose core of the HT. The increase in molecular weight had a smaller effect on the interaction and was easily overshadowed by adding free galloyl groups to the structure. The effect of HHDP groups on the interaction was found to be minor. The newly gained insight from our study strengthens the idea that tannins, along with other natural compounds, could potentiate the effects of anthelmintics simultaneously via both direct and indirect modes of action. However, the in vitro and in vivo applications require their own studies, and the results obtained with TBZ may not be directly translatable to all benzimidazoles or to other anthelmintic classes. 

## Data Availability

The data presented in this study is available on request from the corresponding author.

## Supplementary Materials

The following supporting information can be downloaded at: https://www.mdpi.com/article/10.3390/molecules28135261/s1, Table S1: Hydrolysable tannins used in the study; Figure S1: The effect of reference power values when titrating 3 mM pentagalloylglucose into 3 mM thiabendazole; Figure S2: Raw data and resulting isotherms of the analyzed hydrolysable tannin monomers; Figure S3: Raw data and resulting isotherms of the analyzed hydrolysable tannin dimers; Figure S4: Raw data and resulting isotherm of the analyzed hydrolysable tannin trimer, lambertianin C; Figure S5: Fitted isotherm of the hydrolysable tannin dimer, rugosin D, and corresponding thermodynamic binding parameters.

## Sample Availability

Samples of the compounds used are not available from the authors.
