# Supplementary material for: Tannins Can Have Direct Interactions with Anthelmintics: Investigations by Isothermal Titration Calorimetry
Source: Molecules. 2023 Jul 7;28(13):5261. doi: 10.3390/molecules28135261 (PMC10343783; doi:10.3390/molecules28135261)
Supplement: Supplementary file 1 [file molecules-28-05261-s001.zip › molecules-2450966-supplementary.pdf]

Supplementary material

Tannins Can Have Direct Interactions with Anthelmintics: Investigations by Isothermal Titration Calorimetry

Mimosa Sillanpää [1], Marica T. Engström [1], Petri Tähtinen [1], Rebecca J. Green [2], Jarmo Käpylä [3], Anu Näreaho [4] and Maarit Karonen [1,*]

[1] Department of Chemistry, University of Turku, FI-20014 Turku, Finland; mamsil@utu.fi (M.S.); mtengs@utu.fi (M.T.E.); peppe@utu.fi (P.T.)
[2] School of Chemistry, Food and Pharmacy, University of Reading, Whiteknights, P.O. Box 224, Reading RG6 6AP, UK; rebecca.green@reading.ac.uk
[3] Department of Life Technologies, University of Turku, FI-20014 Turku, Finland; jakapy@utu.fi
[4] Department of Veterinary Biosciences, University of Helsinki, FI-00014 Helsinki, Finland; anu.nareaho@helsinki.fi
* Correspondence: maarit.karonen@utu.fi; Tel.: +358-29-450-3179

Table of Contents

Table S1 2
Figure S1 3
Figure S2 4
Figure S3 5
Figure S4 6
Figure S5 6
References 7

Table S1. Hydrolysable tannins used in the study; the original plant source and material, compound purities, calculated and exact molecular masses and mass error (ppm), the fragmentation patterns used in the identification and the corresponding literature.

No.	Plant source	Plant part	Compound	Purity*	$M_{calculated}$	M_{exact}	M_{error} (ppm)	Fragment ions used in the identification (*m*/*z*)	Literature
**1**	Meadowsweet (*Filipendula ulmaria*)	inflorescence	Tellimagrandin I	97%	786.09060	786.09027	-0.417	785 $[M–H]^–$	[1,2]
**2**	Purple loosestrife (*Lythrum salicaria*)	flowers and leaves	Vescalagin	94%	934.07020	934.06846	-1.861	933 $[M–H]^–$, 915 $[M–H_2O–H]^–$, 466 $[M–2H]^{2–}$, 457 $[M–H_2O –2H]^{2–}$, 301 [ellagic acid–H]$^–$	[2–4]
**3**	Meadowsweet (*Filipendula ulmaria*)	inflorescence	Tellimagrandin II	97%	938.10140	938.09987	-1.629	937 $[M–H]^–$, 301 [ellagic acid–H]$^–$	[1]
**4**	**		1,2,3,4,6-penta-*O*-galloyl-β-D-glucose	99%	940.11700	940.11454	-2.615	939 $[M–H]^–$	[1,5,6]
**5**	Black myrobalan (*Terminalia chebula*)	leaves	Chebulagic acid	96%	954.09630	954.09308	-3.373	953 $[M–H]^–$	[7]
**6**	Black myrobalan (*Terminalia chebula*)	leaves	Chebulinic acid	93%	956.11190	956.11315	1.309	955 $[M–H]^–$	[7]
**7**	Willowherb (*Epilobium angustifolium*)	inflorescence	Oenothein B	95%	1568.15000	1568.14642	-2.280	783 $[M–2H]^{2–}$	[2,3,8]
**8**	Meadowsweet (*Filipendula ulmaria*)	inflorescence	Rugosin E	91%***	1722.17640	1722.17922	1.640	860 $[M–2H]^{2–}$	[9]
**9**	Raspberry (*Rubus idaeus*)	leaves	Sanguiin H-6	93%	1870.15600	1870.15762	0.868	934 $[M–2H]^{2–}$, 301 [ellagic acid–H]$^–$	[3]
**10**	Silverweed (*Argentina anserina*)	leaves	Agrimoniin	97%	1870.15600	1870.15250	-1.869	934 $[M–2H]^{2–}$, 301 [ellagic acid–H]$^–$	[2,3]
**11**	Herb bennet (*Geum urbanum*)	leaves	Gemin A	95%	1872.17160	1872.16876	-1.515	935 $[M–2H]^{2–}$, 301 [ellagic acid–H]$^–$	[3]
**12**	Meadowsweet (*Filipendula ulmaria*)	inflorescence	Rugosin D	91%***	1874.18720	1874.19116	2.115	936 $[M–2H]^{2–}$	[9]
**13**	Raspberry (*Rubus idaeus*)	leaves	Lambertianin C	93%	2804.22620	2804.22510	-0.391	934 $[M–3H]^{3–}$, 301 [ellagic acid–H]$^–$	[3]

* Measured by UPLC-DAD at 280 nm (instrument described in the article, section 3.3.)

** Pentagalloylglucose was prepared via methanolysis [10] from commercial tannic acid purchased from J.T. Baker (Denver, Holland)

*** Rugosins E and D were found to some extent degrade to their monomers during the storage at -20 °C and process of ITC measurements. These purities were determined after the isolation and purification of the rugosins. After the whole ITC study was finalized, the composition of standards was rechecked and the amounts of undecomposable rugosins E and D were found to be 53% and 82% respectively, the main degradation products being the constitutive monomeric units.

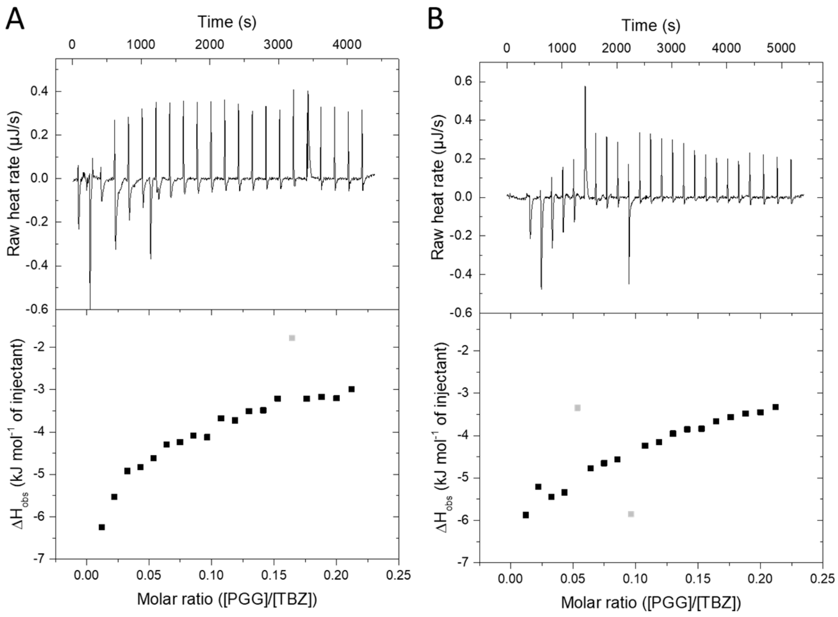

Figure S1. The effect of reference power values of (**A**) 2 µCal/s and (**B**) 5 µCal/s when titrating 3 mM pentagalloylglucose (PGG) into 3 mM thiabendazole (TBZ). The outliers marked in light grey are caused by baseline fluctuation during the titration

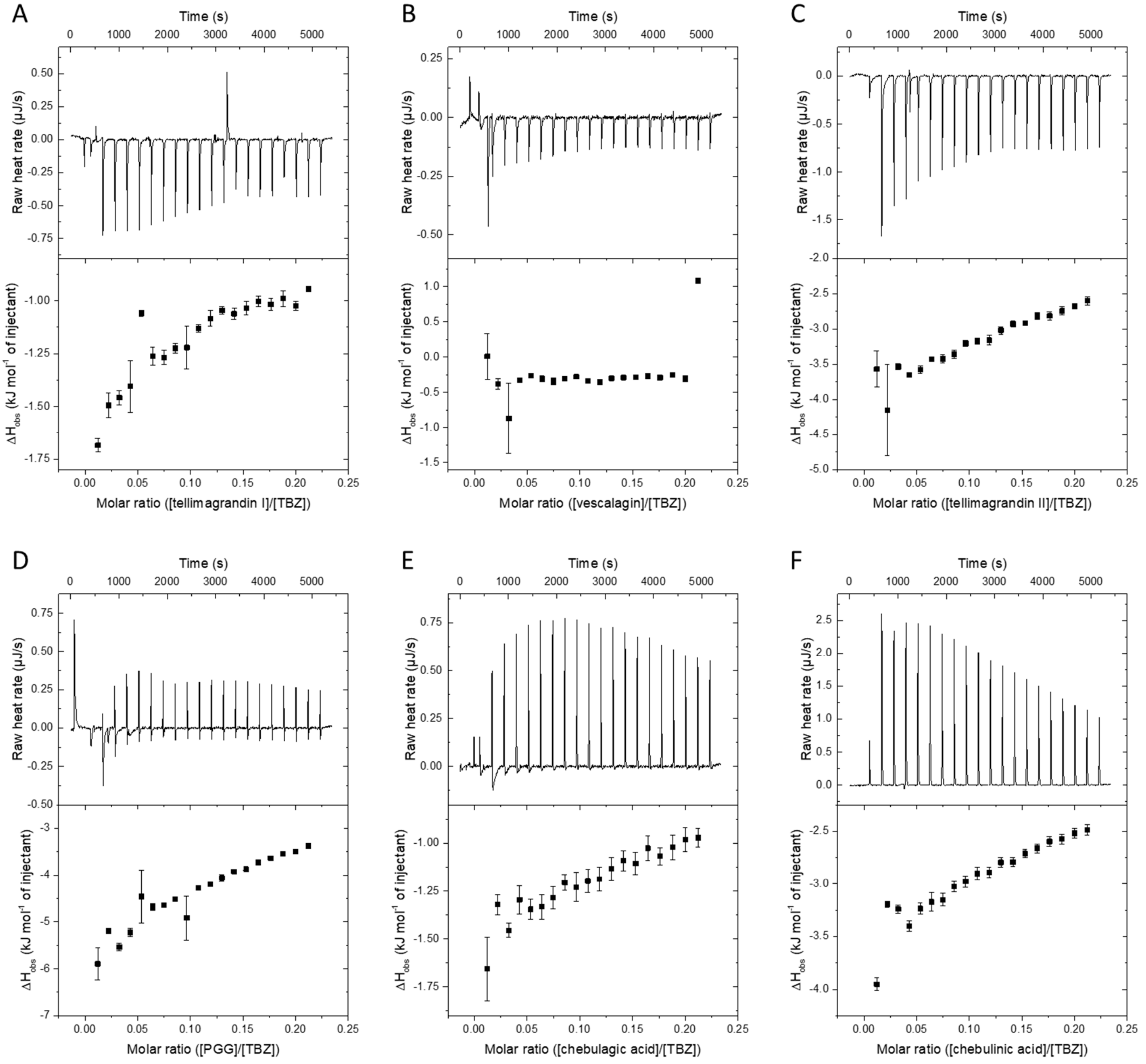

Figure S2. Raw data and resulting isotherms after the integration of peak areas and subtraction of the control measurement (tannin into buffer) of the analyzed hydrolysable tannin (HT) monomers depicting the released heat as kJ per one mole of injectant as a function of molar ratio of tannin to thiabendazole (TBZ). Standard error between three replicates is also shown (n = 3). (**A**) Tellimagrandin I, (**B**) vescalagin, (**C**) tellimagrandin II, (**D**) pentagalloylglucose (PGG), (**E**) chebulagic acid, and (**F**) chebulinic acid. Structures of the HT monomers are presented in the article in Figure 1.

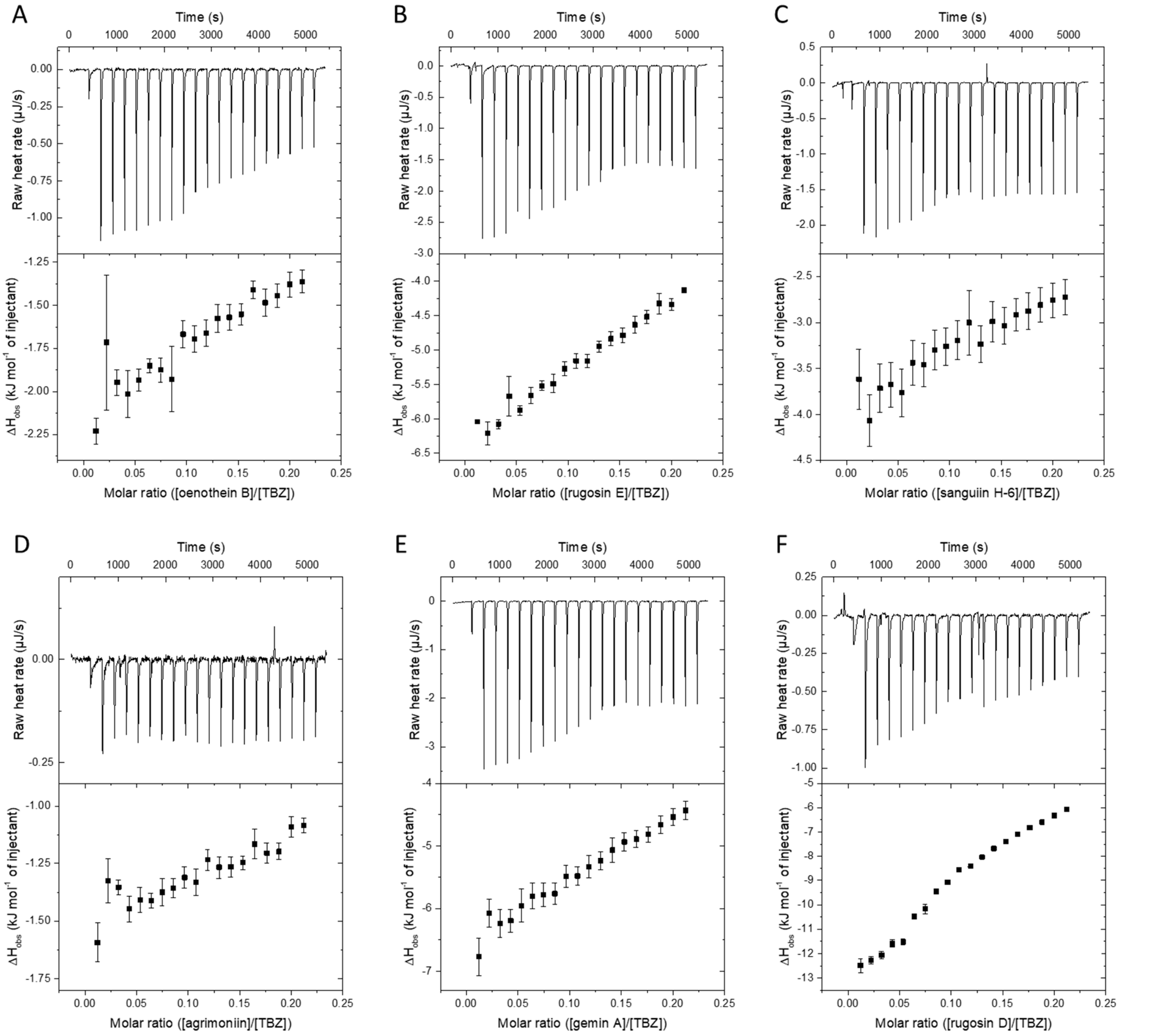

Figure S3. Raw data and resulting isotherms after the integration of peak areas and subtraction of the control measurement (tannin into buffer) of the analyzed hydrolysable tannin (HT) dimers depicting the released heat as kJ per one mole of injectant as a function of molar ratio of tannin to thiabendazole (TBZ). Standard error between three replicates is also shown (n = 3). (**A**) Oenothein B, (**B**) rugosin E, (**C**) sanguiin H-6, (**D**) agrimoniin, (**E**) gemin A, and (**F**) rugosin D. Structures of the HT dimers are presented in the article in Figure 2.

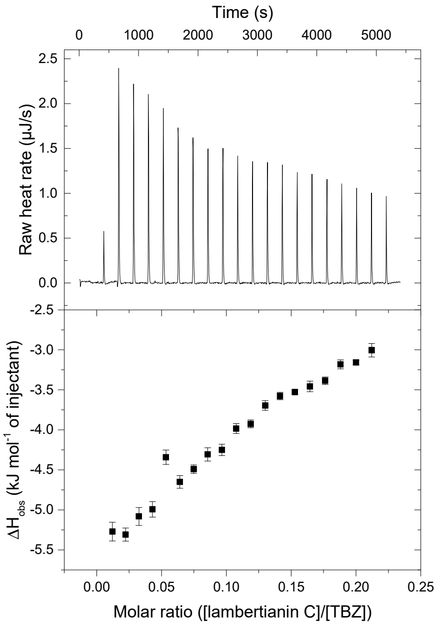

Figure S4. Raw data and resulting isotherm after the integration of peak areas and subtraction of the control measurement (tannin into buffer) of the analyzed hydrolysable tannin (HT) trimer, lambertianin C, depicting the released heat as kJ per one mole of injectant as a function of molar ratio of tannin to thiabendazole (TBZ). Standard error between three replicates is also shown (n = 3). Structure of lambertianin C is presented in the article in Figure 2.

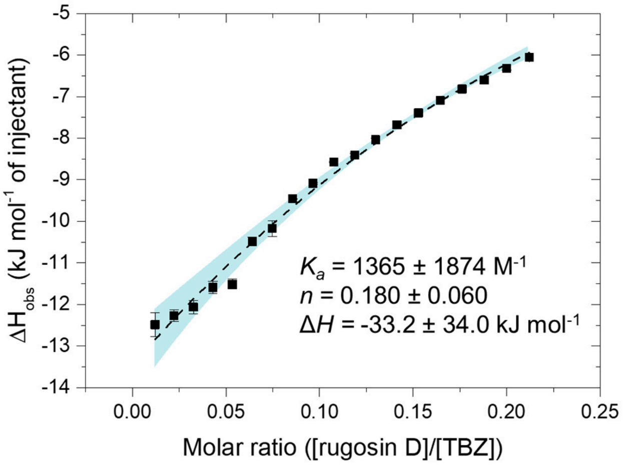

Figure S5. Isotherm of the hydrolysable tannin dimer, rugosin D, fitted with an independent single-site binding model. Each of the three replicates were fitted individually, and the average of these fits is represented by a dashed line. The blue area around the dashed line represents the standard error between the fits. Rough estimates of thermodynamic binding parameters: equilibrium binding constant (K_a), binding stoichiometry (*n*), and enthalpy change (ΔH) and their respective averages of 95 % confidence intervals were obtained by NanoAnalyze software (v. 3.12.0, 2008, TA Instruments).

References

1. Salminen, J.P.; Ossipov, V.; Haukioja, E.; Pihlaja, K. Seasonal Variation in the Content of Hydrolysable Tannins in Leaves of *Betula Pubescens*. *Phytochemistry* **2001**, *57*, 15–22, doi:10.1016/S0031-9422(00)00502-1.
2. Moilanen, J.; Sinkkonen, J.; Salminen, J.-P. Characterization of Bioactive Plant Ellagitannins by Chromatographic, Spectroscopic and Mass Spectrometric Methods. *Chemoecology* **2013**, *23*, 165–179, doi:10.1007/S00049-013-0132-3.
3. Moilanen, J.; Salminen, J.P. Ecologically Neglected Tannins and Their Biologically Relevant Activity: Chemical Structures of Plant Ellagitannins Reveal Their in Vitro Oxidative Activity at High pH. *Chemoecology* **2008**, *18*, 73–83, doi:10.1007/s00049-007-0395-7.
4. Rauha, J.P.; Wolfender, J.L.; Salminen, J.P.; Pihlaja, K.; Hostettmann, K.; Vuorela, H. Characterization of the Polyphenolic Composition of Purple Loosestrife (*Lythrum Salicaria*). *Zeitschrift fur Naturforsch. - Sect. C J. Biosci.* **2001**, *56*, 13–20, doi:10.1515/ZNC-2001-1-203.
5. Salminen, J.P.; Ossipov, V.; Loponen, J.; Haukioja, E.; Pihlaja, K. Characterisation of Hydrolysable Tannins from Leaves of *Betula Pubescens* by High-Performance Liquid Chromatography–Mass Spectrometry. *J. Chromatogr. A* **1999**, *864*, 283–291, doi:10.1016/S0021-9673(99)01036-5.
6. Salminen, J.-P.; Karonen, M. Chemical Ecology of Tannins and Other Phenolics: We Need a Change in Approach. *Funct. Ecol.* **2011**, *25*, 325–338, doi:10.1111/j.1365-2435.2010.01826.x.
7. Pfundstein, B.; El Desouky, S.K.; Hull, W.E.; Haubner, R.; Erben, G.; Owen, R.W. Polyphenolic Compounds in the Fruits of Egyptian Medicinal Plants (*Terminalia Bellerica*, *Terminalia Chebula* and *Terminalia Horrida*): Characterization, Quantitation and Determination of Antioxidant Capacities. *Phytochemistry* **2010**, *71*, 1132–1148, doi:10.1016/J.PHYTOCHEM.2010.03.018.
8. Baert, N.; Karonen, M.; Salminen, J.P. Isolation, Characterisation and Quantification of the Main Oligomeric Macrocyclic Ellagitannins in *Epilobium Angustifolium* by Ultra-High Performance Chromatography with Diode Array Detection and Electrospray Tandem Mass Spectrometry. *J. Chromatogr. A* **2015**, *1419*, 26–36, doi:10.1016/J.CHROMA.2015.09.050.
9. Moilanen, J.; Koskinen, P.; Salminen, J.-P.P. Distribution and Content of Ellagitannins in Finnish Plant Species. *Phytochemistry* **2015**, *116*, 188–197, doi:10.1016/j.phytochem.2015.03.002.
10. Hagerman, A.E.; Rice, M.E.; Ritchard, N.T. Mechanisms of Protein Precipitation for Two Tannins, Pentagalloyl Glucose and Epicatechin16 (4→8) Catechin (Procyanidin). *J. Agric. Food Chem.* **1998**, *46*, 2590–2595, doi:10.1021/jf971097k.
